# Juvenile Dungeness crabs (*Metacarcinus magister*) selectively integrate and modify the fatty acids of their experimental diets

**DOI:** 10.1098/rstb.2020.0038

**Published:** 2020-06-15

**Authors:** Michael D. Thomas, Julie B. Schram, Zade F. Clark-Henry, Bree K. Yednock, Alan L. Shanks, Aaron W. E. Galloway

**Affiliations:** 1 Department of Biology, University of Oregon Institute of Marine Biology, PO Box 5389, Charleston, OR 97420, USA; 2 Department of Forest Ecosystems and Society, Oregon State University College of Forestry, 140 Peavy Hall, 3100 SW Jefferson Way, Corvallis, OR 97331, USA; 3 South Slough National Estuarine Research Reserve, PO Box 5417, Charleston, OR 97420, USA

**Keywords:** Dungeness crab, fatty acids, lipids, trophic modification, diet, biomarkers

## Abstract

Dungeness crabs (*Metacarcinus magister*) are ecologically and economically important in the coastal Northeast Pacific, yet relatively little is currently known about their feeding behaviour in the wild or their natural diet. Trophic biomarkers, such as fatty acids (FA), can be used to reveal trophic interactions. We used two feeding experiments to assess differences in FA composition of juvenile crabs fed different known foods to evaluate how they modify and integrate dietary FA into their own tissues and determine whether crab FA reflect diet changes over a six-week period. These experimental results were then compared with the FA signatures of wild caught juvenile crab with undetermined diets. We found that juvenile Dungeness crabs fed different foods assimilated dietary FA into their tissues and were distinct in their FA signatures when analysed with multivariate statistics. Experimentally fed juvenile crabs contained greater proportions of the most abundant long-chain polyunsaturated fatty acids (LCPUFA, >C20) than their foods. Crabs fed foods lacking in LCPUFA, particularly DHA (22:6*ω*3, docosahexaenoic acid), did not survive or grew slower than crabs fed other foods. This suggests that LCPUFA are physiologically important for this species and indicates biosynthesis of these FA does not occur or is not sufficient to meet their needs.

This article is part of the theme issue ‘The next horizons for lipids as ‘trophic biomarkers’: evidence and significance of consumer modification of dietary fatty acids’.

## 1. Introduction

Determining diets of wild animals is key to understanding organismal ecology and pathways of energy flux through ecosystems [1]. Studying trophic dynamics in aquatic environments is especially challenging because most habitats are difficult to access, making *in situ* observations of feeding activities nearly impossible, especially for cryptic invertebrates. Furthermore, traditional diet studies relying on examinations of gut contents only reflect recently consumed prey and may fail to reflect an animal's complete diet. Thus, many marine ecologists have relied on biomarkers to describe trophic relationships, particularly stable isotopes and fatty acids (FA), which are both useful for integrating dietary information over a longer period than gut content analyses allow. FA have been used as biomarkers in trophic investigations for decades and extensive data are available on the FA signatures of many marine species [2]. FA are particularly useful compared with stable isotopes for discriminating between prey items that might have more variation and less source-specificity than would be observed in isotopic values relative to FA [3]. FA are also useful as biomarkers because their biosynthesis and modification are phylogenetically constrained and certain groups of FA, or multivariate FA composition, may be indicative of specific producers [4–6]. Additionally, there is a general assumption that FA are largely incorporated into consumer tissues with minimal modification, providing a direct link between diet and animal tissues [7]. This direct link can be confounded, however, by modification or selective retention of dietary FA by the consumer, and thus consumer FA profiles will rarely be the same as their prey [7,8]. This has led to an increased focus on the use of feeding experiments to quantify organismal modification and integration of dietary FA.

Researchers often rely on specific ‘biomarker’ FA that are unique or at least characteristic of a particular prey item to infer trophic relationships, but recently, new methods have been developed to quantitatively estimate consumer diets [7,9–12]. These methods use an array of FA (FA ‘signatures’) from consumers and prey to partition the relative contribution of dietary components. For accurate modelling of FA trophodynamics, quantitative techniques require that the analyst account for the consumers' trophic modification of their dietary FA to estimate mixtures of prey items consumed [8–11,13]. FA resource libraries containing the putative prey items are needed, along with the FA signatures of consumers fed known diets. When applying FA techniques to new consumers, long-term, controlled feeding assays using putative diets are needed to understand how consumer lipid metabolism modifies and integrates dietary FA [4,7,8,11].

Dungeness crabs (*Metacarcinus magister*, Dana [14]; formerly *Cancer magister*) are a valuable commercial species and ecologically important predator in the Northeast Pacific [15]. On the West Coast of the USA, commercial landings of Dungeness crabs regularly exceed $200 million per year [16] and the market is entirely dependent on wild caught crabs. Little is known about the diets of wild juvenile crabs. Dungeness crabs are generally known as voracious consumers potentially feeding on an array of taxa, including conspecifics [17], bivalves, fish, polychaetes, shrimps [18,19], and benthic and epiphytic diatoms [20]. High densities of 0+ crabs (young-of-the-year) may have a serious impact on benthic communities and energy transfer out of juvenile habitat. In Oregon, the annual abundance of Dungeness crab larvae fluctuates by a factor of 1000 [21] and periodically large settlement events [22] can result in extremely high densities of juveniles (upwards of tens of thousands m^−2^) in nearshore and estuarine habitats. The settlement of Dungeness crab megalopae in estuarine systems, and subsequent foraging as juvenile crabs, may represent an important link between estuarine and nearshore energy flux as young crabs mature and eventually migrate to the outer coast. Furthermore, density-dependent effects during years of high settlement may regulate annual cohort success and ultimately impact future adult populations [23].

Previous studies of juvenile Dungeness crab diets were based on inspection of stomach contents [18,19], but these methods can be biased to only recently consumed items and prey with easily recognizable hard-parts, and may miss important soft-bodied sources of nutrition [24], especially in very small individuals. Crabs use their chelae and mandibles to tear food into small pieces, further impeding accurate assessment of dietary components. For these reasons, biomarkers such as FA are an attractive solution. There are few studies to date that have examined the FA composition of adult Dungeness crabs [25,26] and none that have explored trophic modification of FA in these consumers.

We used two controlled feeding assays to measure assimilated dietary FA of juvenile Dungeness crabs fed known foods in the laboratory. We also collected wild juvenile Dungeness crabs from multiple locations in an estuary on the southern Oregon coast and compared their FA with crabs from our feeding assays. We compared FA markers in wild crabs with those of experimental crabs for the consumption of copepod (20:1*ω*11) and bacterial (sum of odd/branched-chain FA) prey [4]. Additionally, we use the Σ*ω*3/Σ*ω*6 FA ratio to compare relative proportions of these important groups of FA [27]. Our primary research objectives were to: (1) assess differences in FA composition of crabs fed different known foods (feeding assays 1 and 2); (2) in crabs originally fed the same food, determine if a change in crab FA can be detected six weeks after a change in their diets (assay 1); (3) evaluate how juvenile crabs modify or selectively assimilate and integrate dietary FA of likely *in situ* food sources (assay 2); (4) examine the FA signatures of wild juvenile crabs with undetermined diets and compare them with crabs maintained on known foods in the laboratory (wild crab FA).

## 2. Material and methods

### (a) Feeding assay 1: dietary fatty acids integration

In early June 2017, we sampled wild Dungeness crab megalopae daily using a light trap [28] placed within the Coos Bay estuary approximately 2 km from the estuary mouth (Charleston, OR, USA). Dungeness crab megalopae are pelagic and we have found that using a light trap is an efficient way to sample these larvae. Megalopae were pooled from several days of light-trapping and held in the same container until they all metamorphosed to first instar juveniles (1–6 days post-collection). Juvenile crabs were held together and fed razor clam meat (*Silqua patula*) for two to three weeks until each individual had undergone one moult. We initially maintained crabs on bivalve meat because this is known as an important food for crabs [29] and we needed to maintain the crabs through their metamorphosis from being zooplankton (megalopae) to settled juveniles at similar life stages using a consistent food source. At the end of June, juveniles were separated and placed individually into cylindrical, plastic containers (500 ml) filled with 1 µm filtered seawater (FSW). Air was bubbled through the FSW in each container to maintain adequate levels of dissolved O_2_ and the water was changed daily. Juveniles (*n* = 13 per food) were randomly assigned to one of five food treatments: (1) algae (*Ulva* sp.; collected locally); (2) urchin faeces (*Strongylocentrotus purpuratus* fed *Ulva* sp. in culture); (3) bivalve meat (*S. patula*); (4) megalopae (*M. magister* conspecifics*,* i.e. ‘cannibalism’); and (5) fish meat (*Sebastes melanops*, black rockfish). Urchin faeces derived from macroalgae feed were used to test the possible enriched food value of algae after egestion [30] by urchins. All crab foods were frozen (−20°C) while fresh and chopped into approximately 1 mm pieces before being offered to juveniles, which consumed each food readily. Juvenile crabs were fed these foods ad libitum for six weeks, after which they were starved for one week to purge gut contents, and then frozen and lyophilized. To monitor moult increments, crab carapace widths (CW) were measured photogrammetrically from weekly digital photographs. Unfortunately, we were unable to analyse the FA of the experimental foods used for this experiment, so we focused on the resulting integration and reflection of multivariate FA signatures in crabs fed different diets.

### (b) Feeding assay 2: trophic modification

In late April 2019, we sampled wild Dungeness crab megalopae using the same light trap described above. Megalopae were haphazardly selected and assigned to one of six food treatments (*n* = 8 per treatment). We placed individuals into 1.5 l rectangular plastic containers that were supplied with a constant flow of 5 µm FSW (approx. 1.7 l h^−1^). Seawater temperature and salinity ranged from 11 to 14°C and 31 to 34 PSU, respectively. After megalopae metamorphosed to first instar juveniles, they were held for 11 days without food until the beginning of the experiment.

We collected putative sources of food from the nearby South Slough estuary (arm of larger Coos Bay estuary). Six foods were selected based on their abundance and availability in the estuary at the time of Dungeness crab recruitment: (1) bivalve (mostly *Clinocardum nuttallii*, some juvenile *Macoma* sp*.*); (2) ghost shrimp (*Neotrypaea californiensis*); (3) mysid shrimp (*Neomysis mercedes*); (4) megalopae (*M. magister*); (5) polychaete (*Owenia* spp*.*); and (6) detritus (including sand, organic particulate matter, and any fauna within the upper 1 cm of benthic substratum). In this experiment, we focused on foods that were likely to be included in the diets of crabs in the particular estuary where we were collecting wild crabs. Moreover, because we quantified the FA of both the diets and crabs, we specifically evaluated FA trophic retention and modification in this experiment. Crab foods were frozen while fresh and lyophilized to minimize FA oxidation. Lyophilized tissue was homogenized into a powder using a Vitamix® (Olmstead Falls, OH, USA) blender and then mixed with 2% sodium alginate solution. We homogenized the foods in this assay so that sources with different physical attributes or mixtures would not bias food consumption. We ensured the alginate solution was below 30°C before adding the homogenized powder to prevent thermal degradation of lipids. Most foods were mixed at a concentration of 10% w/v, except polychaete and detritus, which were mixed at 20% w/v because sediments found in these diets resulted in less potential food. The food–alginate solutions were solidified into 300 mg pellets using aqueous 5% CaCl_2_ and stored at −5°C.

We froze excess homogenized food powder and prepared food pellets bi-weekly from these stocks. We haphazardly selected and preserved one food pellet per treatment for FA analysis from each bi-weekly batch and two samples from the remaining food stock at the end of the experiment (*n* = 5 per treatment group). Juvenile crabs were fed ad libitum for six weeks on these six foods, and vigorously consumed all foods, except the detritus, which was more slowly consumed. Prior to daily feeding, uneaten food and crab excrement were removed from each container. At the completion of the experiment, the crabs were starved for one week to purge their stomach contents before being frozen and lyophilized. Crab growth was measured photogrammetrically as described above.

### (c) Wild crab fatty acids

In July and September (2018), we collected wild juvenile Dungeness crabs from three locations within the South Slough estuary in Coos Bay, Oregon (Crown Point, Valino Island, and Sengstacken arm; all within 2 km of each other). Collections were done by beach seining and snorkeling because light traps (used for collecting megalopae for assays 1 and 2) are not effective at targeting benthically associated juveniles. Crown Point and Valino Island are in the marine-dominated middle of the South Slough arm of the Coos Estuary and the Sengstacken site is in the upper estuary, closer to one of the main sources of freshwater input for this arm of the estuary. Juvenile Dungeness crabs were very abundant in 2018 [31]. Haphazardly selected individual crabs (*n* = 4 per site, per month) were held in estuarine water during transport, measured using Vernier calipers, frozen (−20°C), lyophilized until dry (approx. 48 h), and stored at −20°C until lipid extraction. Wild crabs were not starved to eliminate gut contents, so each was split into two parts along the sagittal plane just to the right of the gut and the smaller piece (the part without the stomach, but containing all other tissues) was homogenized for lipid analysis.

### (d) Fatty acid extraction and quantification

Five frozen, dried crabs from each food treatment (seven from *C. nuttallii* treatment group) in both feeding assays 1 and 2 were randomly selected and homogenized whole. An aliquot (10 mg) of homogenized tissue was mixed with chloroform and allowed to sit, refrigerated (−20°C), for at least 24 h to begin cell lysis. Lipid extraction and derivatization of fatty acid methyl esters (FAME) was carried out as in Taipale *et al*. [32] with some modification. Briefly, lipids were extracted using a 2 : 1 : 0.75 mixture of chloroform : methanol : NaCl (0.9%). A known concentration of unmethylated internal standard (19 : 0) was added to each sample at the beginning of the extraction protocol to verify extraction and methylation efficiency. Samples were sonicated and vortexed before the lower organic phase was removed and evaporated to dryness. FAME were formed by adding 1 ml toluene and 2 ml of methanolic sulfuric acid (1%) to dried lipids followed by incubation for 90 min at 90°C. Samples were neutralized with 1.5 ml KHCO_3_ (2%) and thoroughly mixed with 2 ml of hexane. Following centrifugation, the upper layer was removed and hexane evaporated to dryness. The addition of hexane (this time using 1.5 ml) was repeated and the upper layer was again removed, and pooled with the upper layer from the previous step. The dried FAME were diluted in 1.5 ml hexane and stored at −80°C.

FAME were analysed by gas (helium) chromatography and mass spectrometry (Shimadzu GCMS model QP-2020). The GCMS was fitted with an Agilent (Santa Clara, CA, USA) DB-23 high polarity column (30 m × 0.25 mm × 0.15 µm; *L* × ID × film thickness). Column temperature followed Taipale *et al*. [32] to ensure adequate separation of chromatogram peaks (60°C for 1.5 min, heated at 10°C min^−1^ to 100°C, 2°C min^−1^ to 140°C, 1°C min^−1^ to 180°C, and finally heated at 2°C min^−1^ to 210°C and held for 6 min). FA were identified by relative retention times and by examination of mass and specific ions [32]. Peak identifications were checked against a Nu-Check Prep (Elysian, MN, USA) 566C standard.

### (e) Data analyses

Chromatogram peaks of FA were integrated using Shimadzu LabSolutions Insight® software. Peak areas were converted to proportion (% contribution of all identified FA). One-way and two-way permutational analysis of variance (PERMANOVA) (*α* ≤ 0.05, 9999 permutations of raw data, type III sums of squares) in PRIMER v. 6 [33] were used to compare FA profiles by treatments. We used ANOVA tests in R (v. 3.5.1) to make univariate comparisons of individual FA or sums of groups of FA. We used non-metric dimensional scaling (NMDS) to visualize crab FA profiles in multivariate space using the ‘vegan’ package in R [34,35] and plotted the results using the ‘ggplot2’ package [36]. We used arcsine square root transformed proportion data for ANOVAs and multivariate plotting to reduce heteroscedasticity of the data and to improve the interpretability of NMDS plots.

Trophic modification of FA was calculated as the 10% trimmed mean of the ratio between the proportion of FA in consumer tissue to the proportion in their food. These were calculated as in Iverson *et al*. [9] by computing all permutations of ratios between replicate crab tissues and food samples. For example, a food treatment containing five replicate crabs and five replicate samples of the food used in the treatment resulted in 25 ratios per FA, from which the trimmed mean was calculated. Results are presented as mean ± s.d. unless otherwise indicated.

## 3. Results

### (a) Crab fatty acids

A total of 42 FA were identified in wild and experimental juvenile Dungeness crabs. The five most abundant FA made up 75% of the total FA profile (16 : 0, 26.4 ± 2.1%; 20:5*ω*3, 16.6 ± 2.3%; 18 : 0, 13.2 ± 1.6%; 22:6*ω*3, 11.4 ± 2.8%; and 18:1*ω*9, 7.0 ± 1.6%). Saturated FA, monounsaturated FA, polyunsaturated FA, and odd length/branched-chain FA (including 18:1*ω*7) accounted for 43.0 ± 2.1, 11.7 ± 2.0, 35.2 ± 3.3 and 10.0 ± 2.3% of the total FA profile, respectively (electronic supplementary material, tables S1, S2 and S4).

### (b) Feeding assay 1: dietary fatty acids integration

After being isolated and fed a new food for six weeks, all crabs completed at least one additional moult from second to third instar, with some having moulted an additional time to fourth instar. Survival was high in all treatments and averaged 90 ± 10%. The average size increase between second and third instar was 3.2 mm CW (±0.5 mm) and the average increase between third and fourth instar was 4.2 mm CW (±0.7 mm). Crabs fed algae and algae-derived urchin faeces only moulted once and finished at a smaller size (12.0 ± 0.8 mm CW) than those fed the other three foods (15.9 ± 1.6 mm CW).

**Figure 1.**
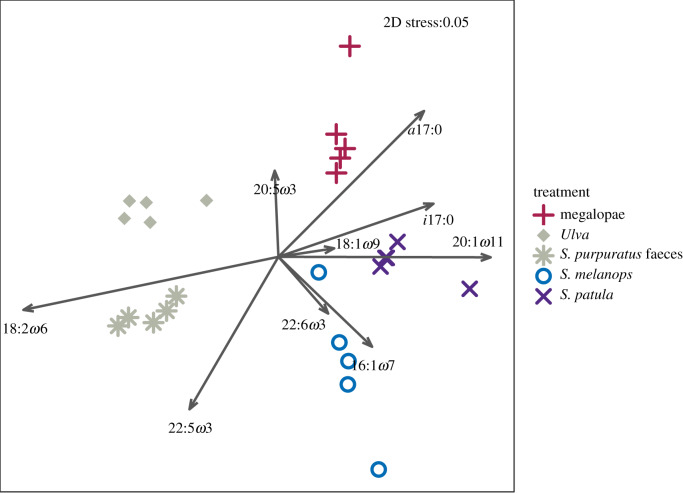
NMDS plot (Euclidean distance) showing arcsine square root transformed proportions of FA (*n* = 42) in juvenile Dungeness crabs (*M. magister*) fed monospecific diets after a food switch (assay 1). Crabs were fed bivalve (razor clam) between first and second instar then fed the foods indicated by symbols for an additional six weeks. (Online version in colour.)

Six weeks after experiencing a food switch, crab FA signatures were all significantly different (one-way PERMANOVA, *post hoc* pairwise comparisons; table 1*a* and electronic supplementary material, table S5) in multivariate NMDS space according to their foods (figure 1). Crabs that did not complete a second moult after the food switch (those fed algae and algae-derived faeces) had the lowest proportion of DHA (22:6*ω*3, docosahexaenoic acid)) and the highest proportions of 18:2*ω*6 (LIN, linoleic acid) and 18:3*ω*3 (ALA, alpha-linoleic acid). Those fed urchin faeces had the lowest *ω*3/*ω*6 ratio (electronic supplementary material, table S1).

**Table 1.** Results of one-way and two-way PERMANOVAs (Euclidean distance) of FA proportion (*n* = 42) from (*a*) crabs in the FA integration experiment (feeding assay 1, pairwise comparison results, electronic supplementary material, table S5), (*b*) juvenile Dungeness crabs fed mono-specific foods in the trophic modification experiment (feeding assay 2, pairwise comparison results, electronic supplementary material, table S6), (*c*) wild juvenile Dungeness crabs caught at three locations in the South Slough estuary in July 2018 and September 2018 (wild crab FA, pairwise comparison results, electronic supplementary material, table S7), and (*d*) wild juvenile Dungeness crabs and laboratory-fed crabs from both feeding assays (combined, pairwise comparison results, electronic supplementary material, table S8).

comparison	variable	d.f.	MS	pseudo-*F*	*p* (perm.)	unique permutations
(*a*) feeding assay 1	treatment	4	0.011	38.133	0.0001	9931
	residual	20	0.0003			
(*b*) feeding assay 2	treatment	7	0.028	56.687	0.0001	9890
	residual	34	0.0005			
(*c*) wild crab FA	location	2	0.006	3.269	0.009	9929
	month	1	0.003	1.511	0.201	9949
	location × month	2	0.004	2.35	0.038	9944
	residual	18	0.002			
(*d*) combined	treatment	9	0.016	13.497	0.0001	9892
	residual	61	0.001

### (c) Feeding assay 2: trophic modification

**Figure 2.**
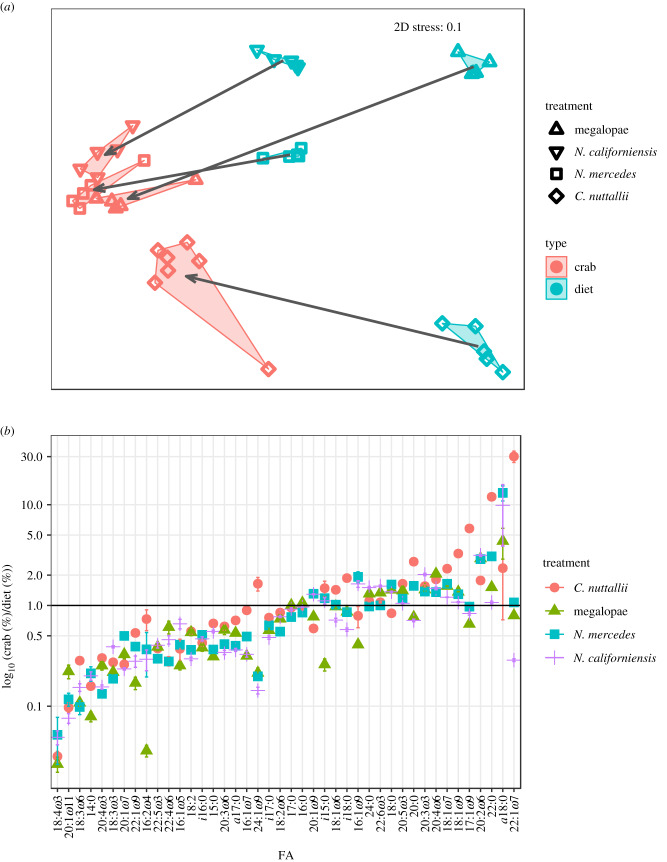
(*a*) MDS plot (Euclidean distance) showing arcsine square root transformed proportions of FA (*n* = 42) in juvenile Dungeness crabs (*M. magister*) and their monospecific diets in the trophic modification experiment (assay 2). Arrows show direction of modification of dietary FA by crabs. Vectors drawn from the approximate centroid of each group of points were added to visualize the multivariate shift in Euclidean distance (similarity) of FA profiles of foods to the resulting crab FA profiles; this shows the relative magnitude and direction of the shift in NMDS space of the crab modification of dietary FA. (*b*) Log_10_ ratio of FA proportion (%) in crab tissues/FA proportion (%) in crab food in the trophic modification experiment, organized by ranking of the ratios. Ratios above 1 (black line) are found in higher proportion in crabs. Error bars are s.e. (Online version in colour.)

Crabs fed the detritus and polychaete foods did not survive and were unavailable for FA analyses. Survival in the remaining treatments averaged 78 ± 6%. Out of 48 juvenile crabs in the trophic modification experiment, only 8% (*n* = 4, three fed bivalve and one fed megalopae) successfully moulted during the six-week experiment. Crabs that moulted increased an average of 2.4 mm ± 0.2 mm CW from first to second instar. Despite not moulting, crabs clearly consumed most foods, and when visualized in multivariate space, FA profiles of crabs fed different foods were distinct from each other and from the FA profiles of their diets (one-way PERMANOVA, *post hoc* pairwise comparisons; table 1*b* and electronic supplementary material, table S6; figure 2*a*). The distance of the trophic modification for bivalves and megalopae was approximately 2× greater than the distance for the ghost shrimp and mysids. When compared with the FA profile of their diets, juvenile crabs preferentially assimilated into their tissues greater proportions of 18:1*ω*9, 18:1*ω*7, and all abundant >C20 polyunsaturated fatty acids (PUFA) (made up at least 1% of total FA), except 22:5*ω*3 (docosapentaenoic acid), which was found in a higher proportion in crab food (figure 2*b*). The proportion of DHA in crabs fed mysid shrimp (14.3 ± 0.7%) was the exception, as it was similar to its food (14.3 ± 0.3%), which had the highest proportion of DHA among all possible crab foods (electronic supplementary material, tables S2 and S3). Most other FA were found to be in greater proportion in crab foods. Compared with other foods, detritus was deficient in long-chain polyunsaturated FA (LCPUFA), most notably EPA (eicosapentaenoic acid, 20 : 5*ω*3) and DHA (electronic supplementary material, table S3). Polychaetes also had the lowest EPA and DHA, but we observed high variability in these values. Both detritus and polychaete diets also had the lowest *ω*3/*ω*6 ratio (electronic supplementary material, table S3).

### (d) Wild crab fatty acids

**Figure 3.**
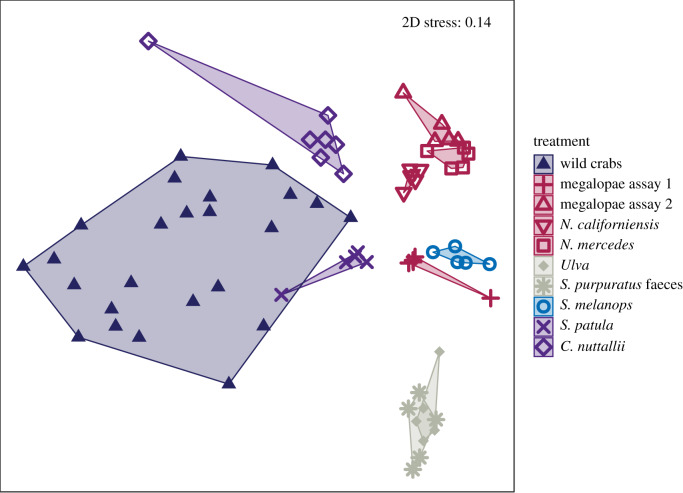
NMDS plot (Euclidean distance) showing arcsine square root transformed proportions of FA (*n* = 42) in wild juvenile Dungeness crabs (*M. magister*) collected in the South Slough estuary (2018) and in crabs fed monospecific diets in two laboratory feeding assays. Polygons are calculated convex hulls for each treatment group. (Online version in colour.)

Wild crabs caught in the South Slough estuary in July 2018 were mostly fifth and sixth instars (20–30 mm CW); those caught in September were seventh instars (approx. 38 mm CW) with one ninth instar (55 mm CW). There was a significant interaction between capture location and time (capture month), and a pairwise comparison (with Monte Carlo permutations) showed that FA profiles of crabs caught at different locations in July were not significantly different, but crabs from Crown Point were significantly different from the other two locations in September (two-way PERMANOVA, *post hoc* pairwise comparisons; table 1*c* and electronic supplementary material, table S7). Wild crab FA profiles clearly differed from those fed most monospecific diets in the laboratory (figure 3). Crabs fed both bivalves (*S. patula* and *C. nuttallii*) were closest in multivariate space to the wild caught crabs, but FA profiles of all laboratory-fed animals differed significantly from those captured in the wild (one-way PERMANOVA, table 1*d* and electronic supplementary material, table S8). Wild crabs had a lower proportion of DHA than all laboratory-fed crabs and were only similar in DHA content to those fed algae and algae-derived faeces (one-way ANOVA, Tukey HSD; *F*_9,61_ = 29.1, *p* < 0.05; electronic supplementary material, table S9). Wild crabs had the highest proportion of copepod indicator FA 20:1*ω*11 (one-way ANOVA, Tukey HSD; *F*_9,61_ = 25.5, *p* < 0.05; electronic supplementary material, table S10). Wild crabs also had the highest sum of bacterial indicator FA (odd length/branched-chain) compared with laboratory-fed crabs and were only similar to those fed bivalves (*S. patula* and *C. nuttallii*), urchin faeces and ghost shrimp (one-way ANOVA, Tukey HSD; *F*_9,61_ = 9.7, *p* < 0.05; electronic supplementary material, table S11).

## 4. Discussion

The FA profiles of juvenile Dungeness crabs fed distinct foods became strongly differentiated in their resulting integrated FA signatures in both feeding assays. Changes occurred over six weeks and even crabs that did not moult assimilated distinctive FA signatures from their diets. Our results are consistent with other studies that indicate marine arthropods' FA can be strongly influenced by their diet, such as in juvenile Chinese horseshoe crabs (*Tachypleus tridentatus*) [37]. When we quantified both food and consumer FA profiles (assay 2), we found that crabs modified dietary FA in a similar way for all experimental diets. This may indicate a consistent strategy for lipid metabolism, which is visualized for individual FA in figure 2*b*. Modification of dietary FA is common in animals [38–41] and not surprising for juvenile Dungeness crabs. Crabs in our experiments were selectively enriched in many LCPUFA when compared with their foods, suggesting that these FA are particularly physiologically important for crustaceans [42–44].

Compared with their foods, laboratory-fed crabs in assay 2 had lower proportions of LCPUFA precursor FA like 18:2*ω*6 (LIN) and 18:3*ω*3 (ALA) and this may suggest that juvenile crabs use these FA to biosynthesize LCPUFA. Most invertebrates have the required biosynthetic pathways for PUFA synthesis [45,46], but without controlled experiments it is unclear if invertebrates can synthesize these FA de novo and if so whether they can do this in biologically relevent amounts to meet physiological needs. The 100% mortality of crabs fed diets (detritus and polychaete) deficient in DHA (22:6*ω*3), however, may indicate that crabs are unable to biosynthesize this FA in sufficient quantities—although there may be other attributes of those foods that caused the mortality, such as overall lower quantities of macronutrients available in each food pellet compared with other treatment groups. Similar to crabs fed detritus and polychaete foods, crabs initially fed bivalve meat before being given macroalgae (*Ulva* sp*.*) and algae-derived urchin faeces also had lower proportions of DHA and grew less than crabs that continued to feed on bivalves, megalopae, or fish meat, and this further suggests the importance of this FA in crab diets.

Juvenile Dungeness crabs significantly modify their dietary FA and selectively assimiliate LCPUFA like DHA, which appears to be an important FA for growth and survival. This is consistent with other studies of crustacean LCPUFA requirements, such as those of juvenile *Panaeus monodon* (tiger prawn), which show significantly increased growth rates when including even small amounts of additional DHA and EPA in LCPUFA-deficient diets [47]. Larval *Scylla serrata* (mud crabs) are also incapable of conversion of LIN and ALA to LCPUFA in significant amounts and showed low survival and extended intermoult periods when fed foods deficient in EPA and DHA [48]. Juvenile *Penaeus chinensis* (Chinese prawn) showed high mortality when fed foods lacking *ω*3 and *ω*6 PUFA and supplemental DHA improved growth and survival more than any other LCPUFA tested [49]. Furthermore, for another kind of marine arthropod, Kwan *et al*. [50] demonstrated the essential role of LCPUFA such as EPA in improving the health and immunocompetency of juvenile Chinese horeshoe crabs.

The wild young-of-the-year (0+) crabs collected in the South Slough estuary in 2018 had the lowest proportion of DHA when compared with laboratory-fed crabs, most simliar to DHA levels of slow-growing crabs fed algae and urchin faeces in assay 1. The observation that wild crabs in 2018 had DHA levels comparable to those that grew the slowest in the laboratory is interesting because Thomas [31] found that age 0+ Dungeness crabs at multiple sites within the same estuary in 2018 grew significantly slower in their first six months post-settlement than they did in the previous 3 years. Dungeness crab larval returns were particulary high in 2018 and were followed by higher summer and autumn densities of 0+ juveniles in the South Slough estuary (2017 individuals ha^−1^) compared with the previous 3 years (119–272 individuals ha^−1^). Water temperatures between the 4 years were not significantly different, and lower crab densities and slower growth in 2018 were hypothesized to have been related to competition for space and resources [31]. It is therefore possible that a DHA limitation due to intense competition for food resulted in slower growth of 0+ crabs in 2018. We have limited information, however, on normal levels of DHA in wild crabs or on how much of this LCPUFA they require.

Allen [25] observed a DHA range of 8.7% (crab viscera) to 14.5% (hepatopancreas) in eight wild adult Dungeness crabs caught near Humboldt Bay, CA. DHA of crabs in our experiments (all tissue types, except stomach) ranged from 7.6 ± 1.3% (wild crabs from Sengstacken) to 16.3 ± 0.6% (crabs fed conspecific megalopae). It is unclear, however, whether FA proportions are consistent between juvenile and adults, as there are no previous FA studies of juvenile Dungeness crabs. Sulkin *et al*. [51] found that zoaeae of Dungeness crabs fed alternating diets of *Artemia* sp. and two dinoflagellates survived as well as those only fed the known high-quality food *Artemia* sp. alone. These dinoflagellates are generally rich in DHA [52] and the importance of this FA for larval crabs may offer insight into its continued importance for juveniles. Future work can address the physiological FA requirements of Dungeness crabs and their ability to biosynthesize LCPUFA, particularly regarding tissue turnover time and how this might change with ontogeny and the slowing moult frequency in older crabs [53].

We found distinct differences between the multivariate FA from wild crabs and those fed putative diets collected from the same habitat (figure 3), suggesting that wild crabs are consuming diets containing additional resources that we did not include in our feeding experiments. In particular, wild crabs contained elevated levels of odd/branched-chain FA and 20:1*ω*11, which suggest bacterial and copepod sources of dietary lipids [4], although 20:1*ω*11 made up only a small proportion (approx. 1%) of total wild crab FA. It is unclear if the wild crabs directly consumed producers of these bacterial and copepod FA or if these lipids were assimilated after eating another consumer. Newly settled wild *Paralithodes camtschaticus* (red king crabs) in Alaska also contained higher proportions of bacterial indicator FA than cultured crabs, and it was hypothesized that naturally occurring biofilms in their habitat may have been the source [54]. Several species of fiddler crabs actively select microorganisms from the sediment and efficiently assimilate bacterial FA into their tissues [55]. Meziane *et al*. suggested these microorganisms were likely an important food source for fiddler crabs [55]. Juvenile *Chionectes bairdi* (tanner crabs) in a sheltered bay in Alaska also had elevated levels of bacterial FA when compared with those found in a more wave-exposed location and this was hypothesized to be related to different sediment characteristics between sites, with the sheltered bay containing more organic-rich sediment [56]. It is also possible that differences in bacterial FA were the result of diet-related differences in gut bacteria composition, although we think this contribution is minimal since we did not include the gut of wild crabs, and crabs in assays 1 and 2 were starved for one week prior to their sacrifice and analysis. Wild crabs were later stage juveniles than experimental crabs and it is possible that some differences in FA composition could be related to ontogenetic differences in how crabs use lipids. Additional work is needed to determine how crab lipid metabolism may change with ontogeny and to extend our research to adult crabs.

The extent that crabs modified their dietary FA was similar among all diet treatments, except for bivalves for some FA (figure 2*b*). Although they appeared to follow a similar pattern of FA modification in this experiment, this may not be the case for crabs consuming other foods. Crabs that obtain a significantly different percentage of their calories from fat compared with our experimental crabs may modify dietary FA in different ways. For example, it is common for animals consuming a high-fat diet to deposit dietary FA directly into lipid stores, but animals consuming lower-fat diets may rely on biosynthesis of FA from dietary carbohydrates and protein to supplement those obtained from their diet [8]. Future work should test crab foods with different levels of lipid and in different amounts [56] to determine if the patterns we observed in trophic modification of dietary FA persist [57]. Additional work is also needed to measure the assimilation of dietary FA from mixed diets rather than just pure diets [41,58,59].

One advantage of using FA in trophic investigations is that organisms integrate dietary information over a longer period of feeding than can be inferred using *in situ* observations or gut content analyses. However, if an animal's diet changes rapidly, this may not be apparent in FA analyses. Young Dungeness crabs grow rapidly, completing several moults in their first six months, and are known to undergo ontogenetic shifts in their diet [19]. They are also highly mobile consumers, capable of migrating to habitats with different available prey items [60], with opportunistic diets including everything from algae [20] to conspecifics (cannibalism; [17]). The FA signatures of crabs fed different foods in this experiment were distinct within six weeks. If FA techniques are going to be applied to wild crabs, it may be prudent to do repeated sampling over time and in multiple locations to elucidate spatio-temporal patterns in FA transfer.

Unique FA that can be attributed to specific sources have been used for decades as biomarkers in trophic investigations, but the field has moved towards the use of multivariate statistics and quantitative models [7,9,11,12]. Our results indicate that quantitative FA analysis, regardless of the exact method used, may be a useful technique for resolving the diets of wild crabs, but they also point to important caveats that must be considered to ensure accurate results. Juvenile Dungeness crabs metabollically modify some components of their dietary FA. Many long-chain (>C20) PUFA and some C18 MUFA are conserved and this must be accounted for in quantitative models. Our results have shown the importance of using controlled feeding assays to understand trophic modification of FA for invertebrate consumers and lay the groundwork for additional study of crustacean diets using FA as biomarkers.

## Supplementary Material

Supplementary Tables S1-11

## Supplementary Material

Data S1 Crab FA Proportion

## Supplementary Material

Data S2 Crab Size

## Data accessibility

The data supporting this article can be found in the figures and tables, and as part of the electronic supplementary material.
