# Supplementary material for: Juvenile Dungeness crabs (Metacarcinus magister) selectively integrate and modify the fatty acids of their experimental diets
Source: Philos Trans R Soc Lond B Biol Sci. 2020 Jun 15;375(1804):20200038. doi: 10.1098/rstb.2020.0038 (PMC7333968; doi:10.1098/rstb.2020.0038)
Supplement: Supplementary Tables S1-11 [file rstb20200038supp1.pdf]

Supplementary materials for:

Juvenile Dungeness crabs (*Metacarcinus magister*) selectively integrate and modify the fatty acids of their experimental diets

Michael D. Thomas, Julie B. Schram, Zade F. Clark-Henry, Bree K. Yednock, Alan L. Shanks, and Aaron W. E. Galloway

Corresponding author: M. Thomas, email: mthomas6@uoregon.edu

Contents:
Tables S1-S11

Other supplementary materials include:
Data S1 Crab FA Proportion (separate file)
Data S2 Crab Size (separate file)

FA Integration Experiment

	Ulva sp.	*S. patula*	*S. purpuratus* feces	*S. melanops*	*M. magister* megalopae
14:0	0.40 ± 0.13	0.46 ± 0.13	0.26 ± 0.03	0.55 ± 0.36	0.23 ± 0.04
16:0	27.41 ± 0.66	25.73 ± 0.64	30.07 ± 0.81	29.23 ± 0.65	24.98 ± 0.42
18:0	12.95 ± 0.3	12.79 ± 0.42	10.87 ± 0.58	12.10 ± 0.75	13.07 ± 0.28
20:0	1.27 ± 0.13	0.93 ± 0.08	1.26 ± 0.08	0.80 ± 0.12	0.83 ± 0.04
22:0	2.10 ± 0.24	0.99 ± 0.12	2.24 ± 0.16	0.88 ± 0.19	1.00 ± 0.08
24:0	0.23 ± 0.01	0.12 ± 0.02	0.21 ± 0.03	0.14 ± 0.02	0.13 ± 0.01
**Σ SFA**	**44.35 ± 0.75**	**41.02 ± 0.57**	**44.92 ± 0.93**	**43.7 ± 0.89**	**40.25 ± 0.31**
16:1ω9	0.05 ± 0.00	0.08 ± 0.02	0.06 ± 0.01	0.13 ± 0.03	0.07 ± 0.01
16:1ω7	1.28 ± 0.16	2.62 ± 0.41	1.31 ± 0.11	2.09 ± 0.72	1.51 ± 0.21
16:1ω5	0.05 ± 0.01	0.07 ± 0.02	0.06 ± 0.01	0.05 ± 0.01	0.07 ± 0.01
17:1ω9	0.14 ± 0.02	0.65 ± 0.10	0.16 ± 0.02	0.25 ± 0.03	0.37 ± 0.05
18:1ω9	7.15 ± 0.80	8.51 ± 0.38	5.98 ± 0.20	9.63 ± 0.69	9.12 ± 0.30
18:1ω6	0.07 ± 0.01	0.27 ± 0.02	0.07 ± 0.00	0.16 ± 0.01	0.32 ± 0.03
20:1ω11	0.03 ± 0.01	0.20 ± 0.09	0.04 ± 0.01	0.06 ± 0.01	0.06 ± 0.01
20:1ω9	0.82 ± 0.04	0.85 ± 0.09	0.77 ± 0.04	0.84 ± 0.11	0.86 ± 0.05
20:1ω7	0.21 ± 0.01	1.17 ± 0.41	0.23 ± 0.02	0.27 ± 0.04	0.39 ± 0.06
22:1ω9	0.14 ± 0.04	0.14 ± 0.02	0.14 ± 0.05	0.08 ± 0.02	0.09 ± 0.01
22:1ω7	0.26 ± 0.02	0.20 ± 0.02	0.25 ± 0.02	0.12 ± 0.00	0.13 ± 0.00
**Σ MUFA**	**10.19 ± 0.93**	**14.76 ± 1.12**	**9.07 ± 0.29**	**13.68 ± 1.56**	**12.99 ± 0.59**
16:2ω4	0.02 ± 0.02	0.02 ± 0.00	0.02 ± 0.01	0.03 ± 0.02	0.02 ± 0.00
18:2ω6	1.88 ± 0.31	0.35 ± 0.01	2.39 ± 0.20	0.59 ± 0.02	0.58 ± 0.02
18:2	0.02 ± 0.01	0.22 ± 0.03	0.04 ± 0.01	0.09 ± 0.02	0.07 ± 0.01
18:3ω6	0.03 ± 0.02	0.01 ± 0.00	0.02 ± 0.00	0.01 ± 0.00	0.01 ± 0.00
18:3ω3	2.89 ± 0.66	0.10 ± 0.01	1.90 ± 0.32	0.12 ± 0.02	0.09 ± 0.01
18:4ω3	0.22 ± 0.05	0.03 ± 0.01	0.11 ± 0.02	0.04 ± 0.03	0.02 ± 0.00
20:2ω6	1.22 ± 0.09	1.18 ± 0.09	1.06 ± 0.09	1.00 ± 0.18	1.44 ± 0.04
20:3ω6	0.08 ± 0.02	0.10 ± 0.02	0.13 ± 0.02	0.06 ± 0.01	0.03 ± 0.00
20:4ω6	2.59 ± 0.15	2.35 ± 0.18	2.74 ± 0.14	1.99 ± 0.22	3.76 ± 0.16
20:3ω3	0.58 ± 0.07	0.16 ± 0.02	0.29 ± 0.03	0.23 ± 0.05	0.19 ± 0.01
20:4ω3	0.17 ± 0.05	0.18 ± 0.02	0.23 ± 0.05	0.12 ± 0.04	0.05 ± 0.00
20:5ω3	16.62 ± 0.84	15.06 ± 1.27	15.79 ± 0.30	14.13 ± 1.63	19.46 ± 0.6
22:4ω6	0.07 ± 0.00	0.26 ± 0.03	0.07 ± 0.01	0.10 ± 0.01	0.12 ± 0.01
22:5ω3	0.97 ± 0.32	0.69 ± 0.09	1.53 ± 0.22	0.94 ± 0.23	0.60 ± 0.05
22:6ω3	10.15 ± 0.21	13.45 ± 0.49	9.84 ± 0.65	15.17 ± 0.64	11.18 ± 0.29
24:1ω9	0.03 ± 0.00	0.02 ± 0.00	0.02 ± 0.01	0.13 ± 0.07	0.02 ± 0.00
**Σ PUFA**	**37.54 ± 0.98**	**34.17 ± 1.57**	**36.19 ± 0.86**	**34.76 ± 1.76**	**37.63 ± 0.59**
i15:0	0.16 ± 0.03	0.21 ± 0.08	0.14 ± 0.04	0.37 ± 0.10	0.14 ± 0.03
15:0	0.48 ± 0.07	0.60 ± 0.08	1.06 ± 0.18	0.37 ± 0.06	0.39 ± 0.04
i16:0	0.00 ± 0.00	0.01 ± 0.00	0.00 ± 0.00	0.01 ± 0.00	0.01 ± 0.00
i17:0	0.31 ± 0.03	0.89 ± 0.18	0.27 ± 0.03	0.55 ± 0.09	0.66 ± 0.10
a17:0	0.08 ± 0.01	0.25 ± 0.05	0.09 ± 0.00	0.12 ± 0.02	0.23 ± 0.04
17:0	0.65 ± 0.03	1.93 ± 0.23	0.75 ± 0.07	0.63 ± 0.11	0.86 ± 0.48
i18:0	0.32 ± 0.06	0.45 ± 0.02	0.28 ± 0.02	0.24 ± 0.06	0.58 ± 0.06
a18:0	0.11 ± 0.01	0.21 ± 0.04	0.09 ± 0.00	0.12 ± 0.02	0.28 ± 0.07
18:1ω7	5.8 ± 0.36	5.50 ± 0.13	7.13 ± 0.28	5.44 ± 0.3	5.99 ± 0.34
**Σ Bacterial**	**7.92 ± 0.4**	**10.05 ± 0.75**	**9.81 ± 0.44**	**7.86 ± 0.31**	**9.13 ± 0.28**
Σ LIN + ALA	4.77 ± 0.96	0.44 ± 0.01	4.29 ± 0.51	0.71 ± 0.04	0.67 ± 0.02
Σ LCPUFA	32.48 ± 0.83	33.45 ± 1.59	31.71 ± 0.72	33.89 ± 1.77	36.85 ± 0.60
ω3/ω6 ratio	5.32 ± 0.20	6.60 ± 0.53	4.59 ± 0.24	7.87 ± 0.48	5.06 ± 0.29

Table S1 – Fatty acid composition of juvenile Dungeness crabs fed bivalve meat for one intermolt cycle and then fed different monospecific foods for six weeks in feeding assay one. Numbers are mean proportion of all FA identified ± SD, except ω3/ω6 which is a ratio of all ω3 FA to all ω6 FA. Rows in bold are the sum of preceding FA (SFA = saturated FA, MUFA = monounsaturated FA, PUFA = polyunsaturated FA, Bacterial = odd/branched chain FA plus 18:1ω7). Σ LCPUFA (long chain PUFA) includes all PUFA with chain lengths > 20. Σ LIN + ALA = Σ 18:2ω6 + 18:3ω3.

Trophic Modification Experiment

	C. nuttallii	*M. magister* megalopae	*N. mercedes*	*N. californiensis*
14:0	0.36 ± 0.15	0.74 ± 0.43	0.46 ± 0.37	0.75 ± 0.38
16:0	24.51 ± 0.65	26.70 ± 0.80	28.64 ± 0.71	25.60 ± 1.07
18:0	12.26 ± 1.01	12.47 ± 0.84	12.74 ± 0.77	14.98 ± 0.37
20:0	0.38 ± 0.05	0.38 ± 0.02	0.51 ± 0.02	0.50 ± 0.05
22:0	0.50 ± 0.12	0.46 ± 0.05	0.63 ± 0.05	0.63 ± 0.08
24:0	1.47 ± 0.13	1.76 ± 0.04	1.64 ± 0.06	1.27 ± 0.13
**Σ SFA**	**39.47 ± 1.67**	**42.52 ± 1.28**	**44.62 ± 0.83**	**43.72 ± 1.29**
16:1ω9	0.09 ± 0.05	0.11 ± 0.03	0.14 ± 0.08	0.15 ± 0.05
16:1ω7	1.89 ± 0.84	1.27 ± 0.44	1.17 ± 0.52	1.70 ± 0.58
16:1ω5	0.05 ± 0.06	0.06 ± 0.03	0.04 ± 0.01	0.13 ± 0.07
17:1ω9	0.43 ± 0.16	0.18 ± 0.04	0.18 ± 0.02	0.18 ± 0.03
18:1ω9	7.19 ± 0.60	7.13 ± 0.33	8.00 ± 0.22	7.79 ± 0.24
18:1ω6	0.24 ± 0.09	0.33 ± 0.07	0.10 ± 0.01	0.26 ± 0.04
20:1ω11	0.30 ± 0.18	0.09 ± 0.07	0.00 ± 0.00	0.02 ± 0.01
20:1ω9	1.00 ± 0.14	0.88 ± 0.19	0.73 ± 0.02	0.70 ± 0.02
20:1ω7	0.71 ± 0.45	0.18 ± 0.09	0.10 ± 0.01	0.16 ± 0.02
22:1ω9	0.02 ± 0.01	0.05 ± 0.03	0.02 ± 0.00	0.03 ± 0.02
22:1ω7	0.07 ± 0.01	0.06 ± 0.01	0.05 ± 0.00	0.07 ± 0.01
**Σ MUFA**	**12.01 ± 2.44**	**10.35 ± 1.07**	**10.55 ± 0.68**	**11.18 ± 0.53**
16:2ω4	0.05 ± 0.06	0.01 ± 0.01	0.08 ± 0.15	0.12 ± 0.13
18:2ω6	0.60 ± 0.04	1.35 ± 0.13	1.25 ± 0.05	0.79 ± 0.08
18:2	0.07 ± 0.02	0.07 ± 0.03	0.04 ± 0.01	0.09 ± 0.02
18:3ω6	0.01 ± 0.00	0.01 ± 0.00	0.01 ± 0.01	0.02 ± 0.01
18:3ω3	0.33 ± 0.05	0.44 ± 0.08	0.26 ± 0.04	0.21 ± 0.02
18:4ω3	0.05 ± 0.03	0.08 ± 0.07	0.04 ± 0.06	0.06 ± 0.05
20:2ω6	1.09 ± 0.07	1.55 ± 0.14	1.25 ± 0.10	0.78 ± 0.09
20:3ω6	0.05 ± 0.02	0.04 ± 0.02	0.02 ± 0.00	0.03 ± 0.01
20:4ω6	2.63 ± 0.24	2.00 ± 0.08	2.59 ± 0.21	2.76 ± 0.33
20:3ω3	0.26 ± 0.03	0.78 ± 0.11	0.43 ± 0.05	0.22 ± 0.01
20:4ω3	0.16 ± 0.06	0.15 ± 0.08	0.06 ± 0.01	0.06 ± 0.01
20:5ω3	17.61 ± 2.27	15.86 ± 1.25	17.18 ± 0.23	19.39 ± 0.77
22:4ω6	0.16 ± 0.10	0.01 ± 0.01	0.01 ± 0.00	0.02 ± 0.01
22:5ω3	0.74 ± 0.31	0.21 ± 0.08	0.15 ± 0.02	0.18 ± 0.05
22:6ω3	13.19 ± 1.18	16.28 ± 0.55	14.37 ± 0.66	10.91 ± 1.27
24:1ω9	0.01 ± 0.00	0.02 ± 0.01	0.02 ± 0.00	0.01 ± 0.00
**Σ PUFA**	**37.02 ± 2.96**	**38.86 ± 0.93**	**37.77 ± 0.8**	**35.63 ± 1.76**
i15:0	0.14 ± 0.13	0.17 ± 0.12	0.14 ± 0.07	0.40 ± 0.14
15:0	0.97 ± 0.31	0.71 ± 0.16	0.36 ± 0.05	0.39 ± 0.05
i16:0	0.41 ± 0.19	0.07 ± 0.03	0.07 ± 0.02	0.09 ± 0.02
i17:0	2.47 ± 0.87	0.35 ± 0.12	0.26 ± 0.04	0.42 ± 0.06
a17:0	0.93 ± 0.38	0.07 ± 0.02	0.10 ± 0.01	0.12 ± 0.01
17:0	1.42 ± 0.09	1.30 ± 0.06	1.37 ± 0.06	1.10 ± 0.10
i18:0	0.50 ± 0.15	0.12 ± 0.03	0.05 ± 0.01	0.13 ± 0.02
a18:0	0.04 ± 0.02	0.02 ± 0.01	0.06 ± 0.02	0.03 ± 0.01
18:1ω7	4.63 ± 0.19	5.45 ± 0.32	4.67 ± 0.35	6.79 ± 0.13
**Σ Bacterial**	**11.51 ± 2.08**	**8.26 ± 0.71**	**7.07 ± 0.31**	**9.47 ± 0.33**
Σ LIN + ALA	0.94 ± 0.05	1.79 ± 0.21	1.51 ± 0.07	1.00 ± 0.10
Σ LCPUFA	35.90 ± 2.98	36.90 ± 1.18	36.08 ± 0.88	34.35 ± 2.01
ω3/ω6 ratio	6.79 ± 0.88	6.41 ± 0.47	6.20 ± 0.22	6.70 ± 0.52

Table S2 – Fatty acid composition of juvenile Dungeness crabs fed different monospecific foods for six weeks in trophic modification experiment (assay 2). Numbers are mean proportion of all FA identified ± SD, except ω3/ω6 which is a ratio of all ω3 FA to all ω6 FA. Rows in bold are the sum of preceding FA (SFA = saturated FA, MUFA = monounsaturated FA, PUFA = polyunsaturated FA, Bacterial = odd/branched chain FA plus 18:1ω7). Σ LCPUFA (long chain PUFA) includes all PUFA with chain lengths > 20. Σ LIN + ALA = Σ 18:2ω6 + 18:3ω3.

Crab Foods – Trophic Modification Experiment

	C. nuttallii	Detritus	*M. magister* megalopae	*N. mercedes*	*N. californiensis*	*Owenia* sp.
14:0	2.21 ± 0.13	3.44 ± 0.43	8.71 ± 0.30	1.99 ± 0.10	3.68 ± 0.28	2.76 ± 0.39
16:0	26.93 ± 2.26	31.13 ± 0.83	25.18 ± 0.61	33.70 ± 0.56	26.55 ± 1.16	29.10 ± 6.13
18:0	14.66 ± 0.81	13.51 ± 8.91	8.73 ± 0.66	7.87 ± 0.91	10.92 ± 0.73	15.18 ± 3.36
20:0	0.14 ± 0.02	1.92 ± 0.35	0.50 ± 0.03	0.33 ± 0.07	0.72 ± 0.10	0.51 ± 0.19
22:0	0.04 ± 0.01	3.87 ± 2.49	0.30 ± 0.02	0.22 ± 0.07	0.59 ± 0.05	0.53 ± 0.05
24:0	1.35 ± 0.06	5.20 ± 3.62	1.35 ± 0.02	1.67 ± 0.04	0.84 ± 0.08	0.62 ± 0.29
**Σ SFA**	**45.33 ± 1.84**	**59.06 ± 1.96**	**44.77 ± 0.37**	**45.78 ± 0.59**	**43.3 ± 0.49**	**48.7 ± 9.59**
16:1ω9	0.23 ± 0.17	0.42 ± 0.11	0.27 ± 0.03	0.07 ± 0.01	0.09 ± 0.02	0.18 ± 0.06
16:1ω7	2.01 ± 0.13	5.42 ± 3.34	3.92 ± 0.08	2.27 ± 0.06	5.02 ± 0.09	3.28 ± 0.32
16:1ω5	0.10 ± 0.01	0.34 ± 0.18	0.22 ± 0.03	0.11 ± 0.01	0.19 ± 0.02	0.15 ± 0.03
17:1ω9	0.08 ± 0.02	1.14 ± 0.63	0.27 ± 0.05	0.18 ± 0.01	0.22 ± 0.02	0.42 ± 0.02
18:1ω9	2.29 ± 0.56	13.41 ± 9.55	5.24 ± 0.16	6.35 ± 0.92	7.25 ± 0.41	4.60 ± 3.42
18:1ω6	0.16 ± 0.02	0.05 ± 0.04	0.34 ± 0.02	0.10 ± 0.01	0.36 ± 0.05	0.12 ± 0.01
20:1ω11	2.90 ± 0.73	0.03 ± 0.02	0.40 ± 0.06	0.05 ± 0.03	0.24 ± 0.03	5.51 ± 1.28
20:1ω9	1.67 ± 0.08	0.18 ± 0.08	1.11 ± 0.06	0.56 ± 0.04	0.54 ± 0.03	0.79 ± 0.12
20:1ω7	2.45 ± 0.38	0.10 ± 0.08	0.53 ± 0.03	0.21 ± 0.02	0.68 ± 0.06	2.55 ± 0.81
22:1ω9	0.04 ± 0.00	0.04 ± 0.02	0.24 ± 0.03	0.06 ± 0.01	0.10 ± 0.01	0.06 ± 0.02
22:1ω7	0.00 ± 0.00	0.10 ± 0.07	0.07 ± 0.01	0.05 ± 0.01	0.23 ± 0.02	0.04 ± 0.03
**Σ MUFA**	**11.94 ± 0.72**	**21.21 ± 5.14**	**12.62 ± 0.15**	**10.01 ± 0.95**	**14.93 ± 0.31**	**17.71 ± 1.49**
16:2ω4	0.06 ± 0.01	0.41 ± 0.24	0.21 ± 0.03	0.16 ± 0.01	0.36 ± 0.04	0.18 ± 0.02
18:2ω6	0.72 ± 0.08	2.59 ± 1.28	1.82 ± 0.05	2.27 ± 0.26	1.02 ± 0.04	1.28 ± 0.70
18:2	0.11 ± 0.01	0.03 ± 0.02	0.13 ± 0.01	0.12 ± 0.01	0.30 ± 0.03	0.06 ± 0.01
18:3ω6	0.04 ± 0.00	0.11 ± 0.05	0.09 ± 0.01	0.06 ± 0.01	0.11 ± 0.01	0.02 ± 0.01
18:3ω3	1.23 ± 0.09	0.94 ± 0.28	1.98 ± 0.08	1.42 ± 0.39	0.54 ± 0.04	0.52 ± 0.14
18:4ω3	1.44 ± 0.19	0.28 ± 0.09	2.78 ± 0.16	0.64 ± 0.23	1.14 ± 0.14	0.15 ± 0.06
20:2ω6	0.62 ± 0.07	0.02 ± 0.02	0.53 ± 0.03	0.44 ± 0.03	0.25 ± 0.03	0.44 ± 0.13
20:3ω6	0.08 ± 0.01	0.05 ± 0.05	0.07 ± 0.02	0.06 ± 0.01	0.10 ± 0.01	0.02 ± 0.00
20:4ω6	1.45 ± 0.14	0.28 ± 0.22	0.97 ± 0.04	1.92 ± 0.22	1.87 ± 0.19	0.97 ± 0.43
20:3ω3	0.17 ± 0.02	0.02 ± 0.01	0.54 ± 0.05	0.32 ± 0.08	0.11 ± 0.01	0.08 ± 0.05
20:4ω3	0.52 ± 0.07	0.07 ± 0.03	0.59 ± 0.04	0.44 ± 0.10	0.36 ± 0.03	0.10 ± 0.05
20:5ω3	10.90 ± 0.57	3.46 ± 2.62	11.41 ± 0.57	14.78 ± 0.70	18.49 ± 0.87	10.47 ± 6.05
22:4ω6	0.52 ± 0.03	0.04 ± 0.06	0.02 ± 0.00	0.03 ± 0.01	0.03 ± 0.01	0.60 ± 0.26
22:5ω3	1.84 ± 0.19	0.10 ± 0.05	0.54 ± 0.09	0.52 ± 0.11	0.43 ± 0.08	2.00 ± 1.17
22:6ω3	12.45 ± 0.69	0.70 ± 0.41	12.13 ± 0.18	14.34 ± 0.32	7.02 ± 0.35	5.49 ± 3.52
24:1ω9	0.01 ± 0.01	0.10 ± 0.05	0.09 ± 0.02	0.12 ± 0.02	0.08 ± 0.01	0.05 ± 0.03
**Σ PUFA**	**32.15 ± 1.21**	**9.18 ± 2.14**	**33.89 ± 0.31**	**37.64 ± 0.48**	**32.22 ± 0.37**	**22.42 ± 11.02**
i15:0	0.08 ± 0.00	1.03 ± 0.60	0.61 ± 0.05	0.12 ± 0.01	0.36 ± 0.05	0.38 ± 0.04
15:0	1.40 ± 0.08	4.98 ± 2.92	2.27 ± 0.14	0.98 ± 0.05	0.69 ± 0.07	2.61 ± 0.17
i16:0	0.88 ± 0.04	0.33 ± 0.15	0.19 ± 0.02	0.13 ± 0.01	0.19 ± 0.02	0.33 ± 0.04
i17:0	3.13 ± 0.33	0.35 ± 0.15	0.59 ± 0.08	0.41 ± 0.04	0.88 ± 0.11	0.97 ± 0.06
a17:0	1.26 ± 0.18	0.29 ± 0.12	0.13 ± 0.01	0.24 ± 0.04	0.34 ± 0.05	0.51 ± 0.03
17:0	1.49 ± 0.16	1.07 ± 0.27	1.29 ± 0.14	1.78 ± 0.09	1.20 ± 0.16	3.61 ± 0.08
i18:0	0.26 ± 0.06	0.04 ± 0.02	0.13 ± 0.01	0.05 ± 0.01	0.23 ± 0.03	0.15 ± 0.01
a18:0	0.06 ± 0.03	0.08 ± 0.07	0.01 ± 0.01	0.01 ± 0.01	0.03 ± 0.03	0.02 ± 0.02
18:1ω7	2.00 ± 0.09	2.38 ± 0.86	3.50 ± 0.06	2.84 ± 0.07	5.64 ± 0.09	2.56 ± 0.15
**Σ Bacterial**	**10.58 ± 0.88**	**10.55 ± 5.00**	**8.71 ± 0.37**	**6.57 ± 0.17**	**9.56 ± 0.41**	**11.17 ± 0.45**
Σ LIN + ALA	1.95 ± 0.07	3.53 ± 1.56	3.79 ± 0.13	3.69 ± 0.65	1.56 ± 0.07	1.80 ± 0.84
Σ LCPUFA	28.55 ± 1.02	4.82 ± 3.27	26.89 ± 0.43	32.96 ± 0.42	28.75 ± 0.50	20.22 ± 11.66
ω3/ω6 ratio	7.96 ± 0.23	2.20 ± 1.50	7.81 ± 0.42	6.72 ± 0.74	7.56 ± 0.73	5.35 ± 2.82

Table S3 – Fatty acid composition of food material fed to juvenile Dungeness crabs in trophic modification experiment (assay 2). Numbers are mean proportion of all FA identified ± SD, except ω3/ω6 which is a ratio of all ω3 FA to all ω6 FA. Rows in bold are the sum of preceding FA (SFA = saturated FA, MUFA = monounsaturated FA, PUFA = polyunsaturated FA, Bacterial = odd/branched chain FA plus 18:1ω7). Σ LCPUFA (long chain PUFA) includes all PUFA with chain lengths > 20. Σ LIN + ALA = Σ 18:2ω6 + 18:3ω3.

	Crown Point		Valino		Sengstacken	
	July	September	July	September	July	September
14:0	2.42 ± 0.54	2.17 ± 0.35	1.79 ± 1.01	0.59 ± 0.37	1.71 ± 1.00	1.16 ± 1.05
16:0	26.57 ± 1.05	26.36 ± 1.31	25.16 ± 0.39	25.15 ± 2.65	26.09 ± 1.93	23.27 ± 0.84
18:0	14.99 ± 1.91	13.58 ± 1.83	12.66 ± 0.41	14.16 ± 1.80	14.27 ± 1.77	16.02 ± 1.79
20:0	0.84 ± 0.08	0.64 ± 0.13	0.69 ± 0.06	0.73 ± 0.09	0.78 ± 0.11	0.87 ± 0.07
22:0	0.91 ± 0.13	0.61 ± 0.07	0.79 ± 0.13	0.93 ± 0.11	0.94 ± 0.21	1.14 ± 0.13
24:0	0.18 ± 0.05	0.11 ± 0.02	1.25 ± 0.23	1.37 ± 0.15	1.19 ± 0.13	1.13 ± 0.10
**Σ SFA**	**45.91 ± 0.49**	**43.47 ± 0.67**	**42.34 ± 0.65**	**42.92 ± 2.07**	**44.98 ± 1.16**	**43.6 ± 1.06**
16:1ω9	0.05 ± 0.01	0.07 ± 0.01	0.05 ± 0.02	0.03 ± 0.01	0.04 ± 0.03	0.04 ± 0.02
16:1ω7	2.49 ± 0.56	3.15 ± 0.56	2.12 ± 0.63	1.23 ± 0.26	2.36 ± 0.87	1.90 ± 0.73
16:1ω5	0.14 ± 0.06	0.14 ± 0.02	0.08 ± 0.04	0.05 ± 0.01	0.10 ± 0.04	0.10 ± 0.06
17:1ω9	0.38 ± 0.14	0.51 ± 0.07	0.44 ± 0.16	0.44 ± 0.33	0.72 ± 0.31	1.22 ± 0.16
18:1ω9	5.50 ± 1.33	4.98 ± 0.43	5.84 ± 0.52	6.59 ± 1.61	5.29 ± 1.03	4.73 ± 0.27
18:1ω6	0.22 ± 0.05	0.22 ± 0.02	0.23 ± 0.02	0.22 ± 0.03	0.22 ± 0.03	0.25 ± 0.04
20:1ω11	0.62 ± 0.24	1.06 ± 0.18	0.91 ± 0.43	0.29 ± 0.07	0.6 ± 0.34	0.69 ± 0.42
20:1ω9	1.15 ± 1.11	0.97 ± 0.42	0.99 ± 0.20	0.71 ± 0.21	0.69 ± 0.17	0.59 ± 0.08
20:1ω7	1.42 ± 0.16	1.99 ± 0.40	1.73 ± 0.60	0.74 ± 0.17	1.31 ± 0.47	1.01 ± 0.48
22:1ω9	0.10 ± 0.07	0.06 ± 0.01	0.07 ± 0.02	0.08 ± 0.08	0.07 ± 0.05	0.07 ± 0.04
22:1ω7	0.30 ± 0.16	0.39 ± 0.10	0.13 ± 0.02	0.09 ± 0.05	0.10 ± 0.02	0.07 ± 0.03
**Σ MUFA**	**12.37 ± 1.47**	**13.54 ± 0.64**	**12.58 ± 1.39**	**10.48 ± 1.86**	**11.51 ± 1.94**	**10.67 ± 1.7**
16:2ω4	0.22 ± 0.15	0.26 ± 0.09	0.09 ± 0.08	0.02 ± 0.01	0.16 ± 0.14	0.11 ± 0.13
18:2ω6	0.92 ± 0.34	0.77 ± 0.11	0.80 ± 0.04	1.06 ± 0.22	1.00 ± 0.49	0.83 ± 0.18
18:2	0.34 ± 0.14	0.26 ± 0.03	0.19 ± 0.04	0.14 ± 0.02	0.18 ± 0.08	0.26 ± 0.09
18:3ω6	0.05 ± 0.02	0.06 ± 0.02	0.03 ± 0.02	0.02 ± 0.01	0.09 ± 0.08	0.05 ± 0.04
18:3ω3	0.64 ± 0.29	0.86 ± 0.36	0.65 ± 0.28	0.58 ± 0.32	0.71 ± 0.33	0.54 ± 0.18
18:4ω3	0.32 ± 0.10	0.60 ± 0.23	0.48 ± 0.38	0.11 ± 0.04	0.37 ± 0.30	0.16 ± 0.14
20:2ω6	0.56 ± 0.10	0.54 ± 0.08	0.92 ± 0.12	0.69 ± 0.11	0.64 ± 0.12	0.56 ± 0.09
20:3ω6	0.10 ± 0.01	0.09 ± 0.02	0.06 ± 0.01	0.06 ± 0.01	0.09 ± 0.04	0.09 ± 0.02
20:4ω6	1.96 ± 0.41	1.74 ± 0.20	1.91 ± 0.19	2.74 ± 0.79	2.45 ± 0.31	3.17 ± 0.62
20:3ω3	0.24 ± 0.06	0.22 ± 0.07	0.33 ± 0.09	0.25 ± 0.03	0.38 ± 0.14	0.24 ± 0.01
20:4ω3	0.23 ± 0.02	0.23 ± 0.06	0.31 ± 0.09	0.15 ± 0.03	0.22 ± 0.05	0.16 ± 0.04
20:5ω3	15.86 ± 1.49	14.75 ± 1.13	14.77 ± 3.82	18.84 ± 2.75	15.78 ± 1.95	16.97 ± 2.8
22:4ω6	0.11 ± 0.03	0.15 ± 0.03	0.22 ± 0.05	0.16 ± 0.02	0.21 ± 0.03	0.17 ± 0.02
22:5ω3	1.20 ± 0.23	1.35 ± 0.19	1.30 ± 0.11	1.10 ± 0.29	1.37 ± 0.48	1.67 ± 0.09
22:6ω3	8.19 ± 1.12	7.68 ± 0.55	9.85 ± 2.00	11.10 ± 1.37	8.68 ± 1.16	7.60 ± 1.30
24:1ω9	0.07 ± 0.04	0.02 ± 0.01	0.04 ± 0.01	0.05 ± 0.07	0.04 ± 0.02	0.03 ± 0.01
**Σ PUFA**	**31.02 ± 1.5**	**29.58 ± 1.19**	**31.95 ± 5.1**	**37.07 ± 2.27**	**32.38 ± 2.2**	**32.62 ± 4.37**
i15:0	0.15 ± 0.07	0.26 ± 0.07	0.22 ± 0.15	0.08 ± 0.02	0.20 ± 0.09	0.18 ± 0.14
15:0	1.16 ± 0.50	1.48 ± 0.20	1.39 ± 0.47	0.83 ± 0.31	1.73 ± 0.79	2.12 ± 0.98
i16:0	0.24 ± 0.09	0.42 ± 0.07	0.41 ± 0.21	0.19 ± 0.05	0.30 ± 0.11	0.37 ± 0.17
i17:0	1.10 ± 0.23	2.46 ± 0.62	2.78 ± 1.33	1.11 ± 0.38	1.33 ± 0.47	1.45 ± 0.36
a17:0	0.55 ± 0.16	1.24 ± 0.29	1.32 ± 0.73	0.52 ± 0.21	0.74 ± 0.34	0.77 ± 0.19
17:0	2.20 ± 0.12	2.51 ± 0.15	2.32 ± 0.35	2.27 ± 0.70	2.92 ± 0.72	4.16 ± 0.43
i18:0	0.07 ± 0.03	0.09 ± 0.02	0.07 ± 0.04	0.09 ± 0.06	0.02 ± 0.02	0.02 ± 0.01
a18:0	0.24 ± 0.05	0.53 ± 0.17	0.57 ± 0.31	0.16 ± 0.08	0.22 ± 0.10	0.21 ± 0.05
18:1ω7	4.98 ± 0.83	4.41 ± 0.31	4.05 ± 0.20	4.27 ± 0.64	3.67 ± 0.35	3.84 ± 0.16
**Σ Bacterial**	**10.70 ± 1.85**	**13.41 ± 1.21**	**13.13 ± 3.42**	**9.53 ± 1.04**	**11.13 ± 1.94**	**13.12 ± 2.12**
Σ LIN + ALA	1.56 ± 0.63	1.63 ± 0.45	1.44 ± 0.30	1.64 ± 0.34	1.70 ± 0.80	1.37 ± 0.35
Σ LCPUFA	28.53 ± 1.71	26.78 ± 1.21	29.72 ± 5.83	35.13 ± 2.63	29.86 ± 3.18	30.66 ± 4.56
ω3/ω6 ratio	6.95 ± 1.17	7.25 ± 0.76	6.65 ± 1.15	6.51 ± 0.46	6.00 ± 1.24	5.35 ± 0.46

Table S4 – Fatty acid composition of wild juvenile Dungeness crabs (n = 4 per group) caught in the South Slough estuary in 2018. Numbers are mean proportion of all FA identified ± SD, except ω3/ω6 which is a ratio of all ω3 FA to all ω6 FA Rows in bold are the sum of preceding FA (SFA = saturated FA, MUFA = monounsaturated FA, PUFA = polyunsaturated FA, Bacterial = odd/branched chain FA plus 18:1ω7). Σ LCPUFA (long chain PUFA) includes all PUFA with chain lengths > 20. Σ LIN + ALA = Σ 18:2ω6 + 18:3ω3.

Groups	t	P(perm)	Unique perms	P(MC)
Algae, Fish	5.6886	0.0102	126	0.0002
Algae, Meg1	6.2661	0.0083	126	0.0001
Algae, BV1	5.4108	0.0087	126	0.0002
Algae, Feces	4.2734	0.0087	126	0.0003
Fish, Meg1	7.0893	0.0073	126	0.0001
Fish, BV1	3.5136	0.0079	126	0.0011
Fish, Feces	6.3571	0.0073	126	0.0001
Meg1, BV1	5.9516	0.0069	126	0.0001
Meg1, Feces	10.374	0.0081	126	0.0001
BV1, Feces	7.6924	0.0078	126	0.0001

Table S5 – Results of pairwise comparisons for one-way PERMANOVA (Euclidean distance) of FA (n = 42) proportion from juvenile crabs fed mono-specific foods in FA integration experiment (assay 1). Food codes are: algae = *Ulva* sp., BV1 = *S. patula*, feces = *S. purpuratus* feces, fish = *S. melanops,* Meg1 = *M. magister* megalopae.

Groups	t	P(perm)	Unique perms	P(MC)
Neo, Meg2	5.6947	0.0075	126	0.0002
Neo, Mys	5.3154	0.0076	126	0.0001
Neo, BV2	3.2223	0.0011	792	0.0009
Neo, FoodBV	8.2313	0.0078	126	0.0001
Neo, FoodMeg	13.627	0.0086	126	0.0001
Neo, FoodMysid	11.011	0.0076	126	0.0001
Neo, FoodNeo	6.2685	0.008	126	0.0001
Meg2, Mys	3.0342	0.0074	126	0.003
Meg2, BV2	3.1036	0.0012	792	0.0014
Meg2, FoodBV	6.9126	0.0081	126	0.0001
Meg2, FoodMeg	11.663	0.007	126	0.0001
Meg2, FoodMysid	8.005	0.0077	126	0.0001
Meg2, FoodNeo	9.2725	0.01	126	0.0001
Mys, BV2	3.333	0.0019	791	0.001
Mys, FoodBV	7.9252	0.0075	126	0.0001
Mys, FoodMeg	15.212	0.0062	126	0.0001
Mys, FoodMysid	8.1445	0.0087	126	0.0001
Mys, FoodNeo	9.3578	0.0085	126	0.0001
BV2, FoodBV	5.7572	0.0011	791	0.0001
BV2, FoodMeg	7.9998	0.0009	791	0.0001
BV2, FoodMysid	7.0925	0.002	790	0.0001
BV2, FoodNeo	5.2906	0.001	792	0.0001
FoodBV, FoodMeg	8.0626	0.0082	126	0.0001
FoodBV, FoodMysid	8.4893	0.0072	126	0.0001
FoodBV, FoodNeo	8.8309	0.0084	126	0.0001
FoodMeg, FoodMysid	13.016	0.0074	126	0.0001
FoodMeg, FoodNeo	11.854	0.0086	126	0.0001
FoodMysid, FoodNeo	11.12	0.0086	126	0.0001

Table S6 – Results of pairwise comparisons for one-way PERMANOVA (Euclidean distance) of FA ($n = 42$) proportion from juvenile crabs fed mono-specific foods in trophic modification experiment (assay 2). Food codes are: BV2 = *C. nuttallii*, Meg2 = *M. magister* megalopae, Mys = *N. mercedes*, Neo = *N. californiensis*. Codes with "Food" prefix were from the foods fed to crabs.

Groups	t	P(perm)	Unique perms	P(MC)
Within level 'July' of factor 'Month'				
Crown Point, Sengstacken	0.87258	0.6819	35	0.5512
Crown Point, Valino	1.3853	0.1736	35	0.1583
Sengstacken, Valino	0.99231	0.3664	35	0.396
Within level 'September' of factor 'Month'				
Crown Point, Sengstacken	2.2664	0.0562	35	0.0269
Crown Point, Valino	2.3876	0.0288	35	0.0122
Sengstacken, Valino	1.7956	0.0284	35	0.0547

Table S7 – Results of pairwise comparisons of interaction term for two-way PERMANOVA (Euclidean distance) of FA (n = 42) proportion from juvenile crabs caught at three locations in the South Slough estuary in July and September 2018.

Groups	t	P(perm)	Unique perms	P(MC)
Wild, Algae	2.6136	0.0001	9531	0.0001
Wild, Fish	4.2945	0.0001	9545	0.0001
Wild, Meg1	3.1525	0.0001	9544	0.0003
Wild, BV1	2.8986	0.0001	9548	0.0001
Wild, Feces	3.5185	0.0001	9508	0.0001
Wild, Neo	2.7691	0.0002	9563	0.0003
Wild, Meg2	3.9134	0.0001	9552	0.0001
Wild, Mys	3.5958	0.0001	9515	0.0001
Wild, BV2	3.0332	0.0001	9923	0.0001
Algae, Fish	5.6886	0.009	126	0.0001
Algae, Meg1	6.2661	0.0057	126	0.0002
Algae, BV1	5.4108	0.0069	126	0.0002
Algae, Feces	4.2734	0.0073	126	0.0002
Algae, Neo	4.6756	0.0069	126	0.0002
Algae, Meg2	6.2184	0.0077	126	0.0001
Algae, Mys	5.8606	0.0068	126	0.0002
Algae, BV2	3.984	0.0017	792	0.0002
Fish, Meg1	7.0893	0.0069	126	0.0001
Fish, BV1	3.5136	0.0076	126	0.0013
Fish, Feces	6.3571	0.0079	126	0.0001
Fish, Neo	6.221	0.007	126	0.0001
Fish, Meg2	3.4883	0.0077	125	0.0009
Fish, Mys	3.5356	0.008	126	0.0015
Fish, BV2	4.2555	0.0014	792	0.0002
Meg1, BV1	5.9516	0.0078	126	0.0001
Meg1, Feces	10.374	0.0067	126	0.0001
Meg1, Neo	3.1112	0.0077	126	0.0011
Meg1, Meg2	7.2414	0.0084	126	0.0001
Meg1, Mys	7.308	0.0082	126	0.0001
Meg1, BV2	3.095	0.0012	792	0.002
BV1, Feces	7.6924	0.0087	126	0.0001
BV1, Neo	5.0725	0.007	126	0.0002
BV1, Meg2	3.8261	0.0084	126	0.0007
BV1, Mys	4.6358	0.0091	126	0.0001
BV1, BV2	2.6067	0.0095	792	0.0061
Feces, Neo	7.3767	0.0084	126	0.0001
Feces, Meg2	7.8226	0.0067	126	0.0001
Feces, Mys	7.7353	0.0089	126	0.0001
Feces, BV2	5.4784	0.0014	792	0.0001
Neo, Meg2	5.6947	0.0082	126	0.0003
Neo, Mys	5.3154	0.0086	126	0.0001
Neo, BV2	3.2223	0.001	792	0.0008
Meg2, Mys	3.0342	0.009	126	0.0025
Meg2, BV2	3.1036	0.0009	792	0.0004
Mys, BV2	3.333	0.0014	791	0.0007

Table S8 – Results of pairwise comparisons of interaction term for one-way PERMANOVA (Euclidean distance) of FA (n = 42) proportion from wild juvenile crabs and laboratory-fed crabs from both feeding assays. Food codes are as in tables S5 and S6.

Groups	Difference	Lower	Upper	p
Meg2-Feces	0.096239	0.053446	0.139032	0
Wild-BV1	-0.07462	-0.10788	-0.04135	0
Wild-BV2	-0.07057	-0.09964	-0.04151	0
Wild-Fish	-0.09914	-0.1324	-0.06588	0
Wild-Meg2	-0.11437	-0.14763	-0.08111	0
Wild-Mys	-0.08784	-0.1211	-0.05458	0
Meg2-Algae	0.091019	0.048226	0.133812	1E-07
Fish-Feces	0.081008	0.038216	0.123801	2.2E-06
Neo-Meg2	-0.07902	-0.12181	-0.03623	3.9E-06
Fish-Algae	0.075788	0.032996	0.118581	1.02E-05
Meg2-Meg1	0.074359	0.031566	0.117151	1.55E-05
Mys-Feces	0.069711	0.026918	0.112504	5.87E-05
Mys-Algae	0.064491	0.021698	0.107284	0.000253
Neo-Fish	-0.06379	-0.10658	-0.021	0.000306
Meg1-Fish	-0.05913	-0.10192	-0.01634	0.001067
Feces-BV2	-0.05244	-0.09206	-0.01282	0.002032
Feces-BV1	-0.05649	-0.09928	-0.01369	0.002115
Neo-Mys	-0.05249	-0.09528	-0.0097	0.00572
Wild-Meg1	-0.04001	-0.07327	-0.00675	0.007308
BV1-Algae	0.051266	0.008473	0.094059	0.007681
BV2-Algae	0.047221	0.007602	0.086839	0.008174
Mys-Meg1	0.047831	0.005038	0.090624	0.017031
Meg2-BV2	0.043798	0.00418	0.083417	0.019148
Wild-Neo	-0.03535	-0.06861	-0.00209	0.028572
Meg2-BV1	0.039753	-0.00304	0.082546	0.09008
Neo-BV1	-0.03927	-0.08206	0.003527	0.098517
Neo-BV2	-0.03522	-0.07484	0.004398	0.122601
Meg1-BV1	-0.03461	-0.0774	0.008187	0.215491
Meg1-BV2	-0.03056	-0.07018	0.009058	0.272484
Fish-BV2	0.028568	-0.01105	0.068186	0.362938
Wild-Algae	-0.02335	-0.05661	0.009912	0.400878
Mys-Meg2	-0.02653	-0.06932	0.016265	0.578351
Fish-BV1	0.024523	-0.01827	0.067315	0.680981
Wild-Feces	-0.01813	-0.05139	0.015132	0.738871
Meg1-Feces	0.02188	-0.02091	0.064673	0.802698
Mys-BV2	0.017271	-0.02235	0.056889	0.912355
Neo-Feces	0.01722	-0.02557	0.060012	0.944764
Meg1-Algae	0.01666	-0.02613	0.059453	0.954783
Meg2-Fish	0.015231	-0.02756	0.058023	0.974414
Mys-BV1	0.013225	-0.02957	0.056018	0.990238
Neo-Algae	0.012	-0.03079	0.054792	0.995174
Mys-Fish	-0.0113	-0.05409	0.031495	0.996927
Feces-Algae	-0.00522	-0.04801	0.037573	0.999995
Neo-Meg1	-0.00466	-0.04745	0.038132	0.999998
BV2-BV1	-0.00405	-0.04366	0.035573	0.999999

Table S9 – Results of pairwise comparisons of one-way ANOVA (Tukey HSD) of DHA (22:6ω3) from wild juvenile crabs and laboratory-fed crabs from both feeding assays. Food codes are as in tables S5 and S6.

Groups	Difference	Lower	Upper	p
Wild-Algae	0.06263	0.037602	0.087659	0
Wild-Feces	0.060887	0.035858	0.085916	0
Wild-Fish	0.056482	0.031453	0.081511	0
Wild-Meg1	0.055098	0.030069	0.080127	0
Wild-Mys	0.073571	0.048542	0.098599	0
Wild-Neo	0.066773	0.041744	0.091801	0
Wild-Meg2	0.051044	0.026016	0.076073	3E-07
Mys-BV2	-0.04635	-0.07616	-0.01653	0.000144
Wild-BV1	0.035879	0.01085	0.060908	0.000593
Neo-BV2	-0.03955	-0.06936	-0.00974	0.001968
Wild-BV2	0.027224	0.005354	0.049095	0.004733
BV2-Algae	0.035406	0.005594	0.065218	0.008533
Mys-BV1	-0.03769	-0.06989	-0.00549	0.010139
Feces-BV2	-0.03366	-0.06347	-0.00385	0.015246
Fish-BV2	-0.02926	-0.05907	0.000554	0.058606
Neo-BV1	-0.03089	-0.06309	0.001307	0.070421
Meg1-BV2	-0.02787	-0.05769	0.001938	0.085867
BV1-Algae	0.026752	-0.00545	0.058952	0.185955
Meg2-BV2	-0.02382	-0.05363	0.005992	0.229396
Feces-BV1	-0.02501	-0.05721	0.007192	0.263883
Mys-Meg2	-0.02253	-0.05473	0.009674	0.40584
Fish-BV1	-0.0206	-0.0528	0.011597	0.534078
Meg1-BV1	-0.01922	-0.05142	0.012981	0.629403
Mys-Meg1	-0.01847	-0.05067	0.013728	0.679647
Mys-Fish	-0.01709	-0.04929	0.015112	0.76696
Neo-Meg2	-0.01573	-0.04793	0.016472	0.841155
Meg2-BV1	-0.01517	-0.04737	0.017035	0.867605
Mys-Feces	-0.01268	-0.04488	0.019517	0.951444
Neo-Meg1	-0.01167	-0.04388	0.020526	0.97114
Meg2-Algae	0.011586	-0.02061	0.043787	0.972529
Mys-Algae	-0.01094	-0.04314	0.02126	0.981209
Neo-Fish	-0.01029	-0.04249	0.02191	0.987648
Meg2-Feces	0.009843	-0.02236	0.042043	0.990972
BV2-BV1	0.008655	-0.02116	0.038466	0.993775
Meg1-Algae	0.007533	-0.02467	0.039733	0.998791
Neo-Mys	0.006798	-0.0254	0.038998	0.999466
Fish-Algae	0.006148	-0.02605	0.038349	0.999764
Neo-Feces	-0.00589	-0.03809	0.026315	0.999836
Meg1-Feces	0.005789	-0.02641	0.03799	0.999857
Meg2-Fish	0.005438	-0.02676	0.037638	0.999915
Fish-Feces	0.004405	-0.0278	0.036606	0.999986
Neo-Algae	-0.00414	-0.03634	0.028058	0.999992
Meg2-Meg1	0.004054	-0.02815	0.036254	0.999993
Feces-Algae	0.001743	-0.03046	0.033944	1
Meg1-Fish	0.001384	-0.03082	0.033584	1

Table S10 – Results of pairwise comparisons of one-way ANOVA (Tukey HSD) of copepod indicator (20:1ω11) from wild juvenile crabs and laboratory-fed crabs from both feeding assays. Food codes are as in tables S5 and S6.

Groups	Difference	Lower	Upper	p
Wild-Mys	0.080643	0.039694	0.121592	8E-07
Wild-Fish	0.06546	0.024511	0.106409	8.49E-05
Wild-Algae	0.064435	0.023486	0.105383	0.000115
Mys-BV2	-0.07611	-0.12488	-0.02734	0.000134
Wild-Meg2	0.058252	0.017303	0.099201	0.000672
Fish-BV2	-0.06093	-0.1097	-0.01215	0.00452
BV2-Algae	0.059902	0.011127	0.108676	0.005632
Meg2-BV2	-0.05372	-0.10249	-0.00494	0.019915
Wild-Meg1	0.042648	0.001699	0.083597	0.03467
Mys-BV1	-0.05345	-0.10613	-0.00077	0.044032
Mys-Feces	-0.04954	-0.10222	0.003141	0.082228
Wild-Neo	0.036926	-0.00402	0.077875	0.111323
Neo-Mys	0.043717	-0.00897	0.096399	0.18718
Meg1-BV2	-0.03812	-0.08689	0.010659	0.256183
Wild-Feces	0.031101	-0.00985	0.07205	0.292452
Fish-BV1	-0.03827	-0.09095	0.014413	0.352641
Mys-Meg1	-0.03799	-0.09068	0.014688	0.362693
BV1-Algae	0.037244	-0.01544	0.089926	0.390865
Neo-BV2	-0.03239	-0.08117	0.016381	0.480694
Wild-BV1	0.027191	-0.01376	0.06814	0.480959
Fish-Feces	-0.03436	-0.08704	0.018323	0.506797
Feces-Algae	0.033333	-0.01935	0.086016	0.549859
Meg2-BV1	-0.03106	-0.08374	0.021621	0.645311
Feces-BV2	-0.02657	-0.07534	0.022206	0.739567
Neo-Fish	0.028534	-0.02415	0.081216	0.745703
Neo-Algae	0.027508	-0.02517	0.080191	0.783025
Meg2-Feces	-0.02715	-0.07983	0.025531	0.795435
BV2-BV1	0.022658	-0.02612	0.071432	0.87664
Meg1-Fish	0.022812	-0.02987	0.075494	0.915569
Mys-Meg2	-0.02239	-0.07507	0.030291	0.923995
Meg1-Algae	0.021786	-0.0309	0.074468	0.935092
Neo-Meg2	0.021326	-0.03136	0.074008	0.94277
Mys-Algae	-0.01621	-0.06889	0.036474	0.990547
Meg2-Meg1	-0.0156	-0.06829	0.037079	0.992801
Meg1-BV1	-0.01546	-0.06814	0.037225	0.993276
Mys-Fish	-0.01518	-0.06786	0.0375	0.994099
Meg1-Feces	-0.01155	-0.06423	0.041135	0.999278
Neo-BV1	-0.00974	-0.06242	0.042947	0.99982
Meg2-Fish	0.007208	-0.04547	0.059891	0.999986
Wild-BV2	0.004533	-0.03125	0.040315	0.999993
Meg2-Algae	0.006183	-0.0465	0.058865	0.999996
Neo-Feces	-0.00583	-0.05851	0.046857	0.999998
Neo-Meg1	0.005722	-0.04696	0.058404	0.999998
Feces-BV1	-0.00391	-0.05659	0.048772	1
Fish-Algae	-0.00103	-0.05371	0.051656	1

Table S11 – Results of pairwise comparisons of one-way ANOVA (Tukey HSD) of bacterial indicator (odd length/branched chain plus 18:1ω7) from wild juvenile crabs and laboratory-fed crabs from both feeding assays. Food codes are as in tables S5 and S6.
